# The prevalence and outcomes of pT0 disease after neoadjuvant hormonal therapy and radical prostatectomy in high-risk prostate cancer

**DOI:** 10.1186/s12894-015-0079-5

**Published:** 2015-08-13

**Authors:** Jae Young Joung, Jeong Eun Kim, Sung Han Kim, Ho Kyung Seo, Jinsoo Chung, Weon Seo Park, Eun Kyung Hong, Kang Hyun Lee

**Affiliations:** Center for Prostate Cancer, National Cancer Center, Goyang, South Korea; Department of Pathology, National Cancer Center, Goyang, South Korea

## Abstract

**Background:**

To identify the prevalence and clinical outcomes of pT0 disease following neoadjuvant hormonal therapy (NHT) and radical prostatectomy (RP) in high-risk prostate cancer.

**Methods:**

We retrospectively included 111 patients who had received NHT and RP for the treatment of high-risk prostate cancer. We classified the patients into two groups, the pT0 group and the non-pT0 group, depending on whether a residual tumor was observed.

**Results:**

We identified 6 cases (5.4 %) with pT0 disease after reviewing the slides of all patients. There was no recurrence of disease in the pT0 group during a median follow-up of 59 months. Among the 105 patients in the non-pT0 group, biochemical recurrence (BCR) developed in 60 patients (57.1 %), with the median time to BCR being 14 months.

**Conclusions:**

Among the 111 patients with high-risk prostate cancer, we found 6 cases that showed a complete pathological response after NHT and no recurrence of disease during the follow-up, meaning that the androgen deprivation therapy could potentially eradicate high-risk prostate cancer. This is one of the largest studies demonstrating the prevalence of pT0 disease and its outcomes after NHT among patients with high-risk prostate cancer.

## Background

pT0 prostate cancer, also known as no residual tumor, undetectable tumor, or vanishing tumor, is not common in biopsy-proven cases of prostate cancer. In patients who have not received androgen deprivation therapy (ADT) prior to radical prostatectomy (RP), the incidence of pT0 disease is reported to be between 0.07 and 0.5 % [1, 2]. In reality, it is difficult for the surgeon to explain the final diagnosis of pT0 disease to patients, due to their concerns regarding unnecessary surgery for insignificant disease. Conversely, in patients with advanced stage disease such as high-risk disease or locally advanced disease, we suggest that a diagnosis of pT0 following ADT could indicate a complete pathological response or complete tumor eradication after neoadjuvant hormonal therapy (NHT). pT0 disease is more common in patients who have received ADT, and has a wider prevalence than in patients who have not received ADT.

To date, the use of ADT prior to RP has been explored in multiple studies [3]. However, the use of ADT in a neoadjuvant setting prior to RP is not currently recommended due to the absence of clear evidence of a survival benefit of ADT for localized prostate cancer. Considering only high-risk disease or locally advanced disease, ADT followed by RP achieves long-term progression-free survival (PFS) and overall survival (OS) comparable to alternative strategies such as combined radiation therapy (RT) and ADT [4, 5].

At our institution, we perform ADT prior to RP in patients with locally advanced prostate cancer or high-risk disease at the surgeon’s discretion. Notably, we have experienced pT0 disease following NHT and RP among these patients. We designed this study to confirm our hypothesis that pT0 disease following NHT and RP could show outstanding clinical outcomes in patients with high-risk disease. There exist rare reports in the literature that demonstrate the occurrence of pT0 disease in high-risk prostate cancer through subgroup analysis in a small number of patients [6, 7]. Here, we review our prostate cancer database to identify the prevalence of pT0 disease among patients with high-risk disease and the clinical outcomes of these patients during the follow-up. To the best of our knowledge, this is one of the largest studies to demonstrate the prevalence of pT0 disease after NHT in more than 100 patients belonging to the high-risk group.

## Methods

### Patient selection

Between May 2002 and June 2013 we retrospectively identified 120 patients who had received NHT and RP by reviewing the Korean National Cancer Center’s prostate cancer database, in which the clinicopathological data and clinical outcomes were prospectively recorded. In each case, the combination of NHT and surgery was decided at the surgeon’s discretion. Among these 120 patients, we selected 111 patients who had high-risk prostate cancer that was defined based on the D’Amico criteria as PSA ≥ 20 ng/ml, biopsy Gleason score (GS) ≥8, or clinical stage ≥ cT2c for the present study. After obtaining informed consent from each patient, NHT and RP were performed. NHT consisted of at least 3 months of luteinizing hormone-releasing hormone (LHRH) agonist therapy, and radical prostatectomy was performed with standard pelvic lymph node dissection. All of the patients were followed up with serum PSA evaluation every 3 months for the first year, biannually from the second to the fifth year, and annually thereafter. When biochemical recurrence (BCR) developed, radiological studies such as magnetic resonance imaging, computed tomography, or bone scanning were performed, if required. When the (PSA) nadir level did not decrease below 0.2 ng/ml following RP, adjuvant ADT or RT was given at the physician’s discretion. Salvage RT or ADT was recommended for the patients who experienced local recurrence or distant metastasis.

For pathological diagnosis, all of the prostatectomy specimens were processed using complete transverse sections of the whole mounted prostatectomy specimens, from the apex to the base at 4-mm intervals. In addition to conventional hematoxylin & eosin (H&E) staining, we performed immunohistochemical staining in all the cases using several antibodies including PSA, PSMA, PSCA, AMACR, and p63. For the cases that were recorded as having pT0 disease in our database, two pathologists reviewed all the slides in each case in order to confirm the pT0 stage. This study was approved by the Institutional Review Board of the Korean National Cancer Center (NCCNCS05-049). Subject’s informed consent was obtained and documented in accordance with local regulations, ICH-GCP requirements, and the ethical principles that have their origin in the principles of the Declaration of Helsinki.

### Statistical analysis

For comparative analysis, we classified the patients into two groups, the pT0 group and the non-pT0 group depending on whether a residual tumor was observed. We compared the clinicopathological characteristics between the two groups using the Fisher’s exact test for categorical variables, and the Wilcoxon rank-sum test for continuous variables. BCR definition that we currently use after RP is 2 consecutive values of 0.2 ng/ml or greater after surgery. Local recurrence was defined as a newly developed lesion identified around the area of prior prostatectomy in any of the imaging studies. Clinical outcomes of patients after treatment were determined based on BCR or clinical progression, including local recurrence, distant metastasis, and survival, by applying the Kaplan-Meier method. PFS was defined as the time to the first evidence of biochemical, local, or distant metastasis, or death in the absence of disease progression. Statistical significance was defined as *p* < 0.05. All analyses were performed using the SPSS software package (SPSS, Inc., Chicago, version 12.0 for Windows 2007).

## Results

Among the 111 patients with high risk disease, we identified 6 cases (5.4 %) with pT0 disease in our study. After reviewing all the slides of these 6 cases, we did not find any apparent tumors, hence we finally confirmed the presence of pT0 disease. Microscopic findings in the prostate tissue after ADT included atrophy of the gland, basal cell prominence, vacuolated luminal cell layers, and squamous and transitional cell metaplasia of the non-tumor gland. The tumor glands usually showed smaller tumors within, pyknosis, empty glandular spaces, and vacuolization and degeneration of tumor cells with an inflammatory response. When pyknotic cells were positive for cytokeratin, PSA, or AMACR on immunohistochemical staining, they were considered to demonstrate the presence of a viable residual tumor.

Patient characteristics of the 6 cases of pT0 disease are presented in Table 1. Patient age was significantly different between the pT0 and the non-pT0 groups. The 6 patients with pT0 disease were older than the patients with non-pT0 disease (*p* = 0.014). Otherwise, no significant differences were observed in the clinical features including the baseline PSA level, biopsy GS, clinical stage, or number of positive biopsy cores (Table 2).

**Table 1:** Characteristics of patients with pT0 prostate cancer

Patient number	Age	PSA (ng/mL))		Duration of NHT	Clinical stage	GS	Number of positive cores on biopsy	Maximal tumor length on biopsy (mm)	Duration of follow-up
		Baseline	After NHT						
1	66	24.6	<0.1	6 months	T2cNoMo	7	6	10.0	48
2	77	55.3	0.3	3 months	T3aN0M0	6	3	3.0	60
3	66	20.9	<0.1	6 months	T3aN0M0	7	3	21.0	80
4	76	6.18	<0.1	3 months	T2cNoMo	7	8	12.0	74
5	73	9.4	<0.1	12 months	T3aN0M0	7	8	5.0	57
6	74	19.38	<0.1	6 months	T3aN0M0	8	6	10.0	6

*PSA* prostate-specific antigen, *NHT* neoadjuvant hormonal therapy, *GS* Gleason score

**Table 2:** Clinicopathological features between the two groups

	pT0 group (*n* = 6)	Non-pT0 group (*n* = 105)	*P*-value
Age, median (range)	73.5 (66–77)	66.0 (47–77)	0.014
PSA, median (range)	20.14 (6.18–55.30)	39.50 (4.16–261.80)	0.126
Biopsy GS (%)			0.447
2–6	1 (16.7)	19 (18.1)	
7	4 (66.7)	44 (41.9)	
8–10	1 (16.7)	42 (40.0)	
Clinical stage (%)			0.461
cT1c- cT2b	0 (0.0)	10 (9.5)	
cT2c	2 (33.3)	50 (47.6)	
cT3-cT4	4 (66.7)	45 (42.9)	
Median number of positive cores on biopsy (range)	6 (3–8)	6 (1–13)	0.599
Median maximum tumor length (range)	10.0 mm (3.0–21.0)	11.0 mm (0.5–25.0)	0.806
Median duration of NHT (range)	6 months (3–12)	3 months (3–30)	0.144
Median (PSA) nadir after NHT (range)	0.01 ng/ml (0.01–0.30)	0.20 ng/ml (0.01–23.70)	0.387
Pathological stage (%)			
pT0	6 (100.0)		
pT2		46 (43.8)	
pT3-pT4		46 (43.8)	
N1		13 (12.4)	
Positive resection margin		28 (26.7)	
In pT2		7 (15.2 %)	
In pT3-pT4		15 (32.6 %)	
In N1		6 (46.2 %)	
Seminal vesicle invasion		36 (34.3))	
Prostate volume, median (range)	22.0 gm (16.0–24.1)	24.9gm (7.5–50.0)	0.280
Tumor volume, % (range)	0	20 (1–90)	

*PSA* prostate-specific antigen, *NHT* neoadjuvant hormonal therapy, *GS* Gleason score, *NA* not available

Regarding the pathological stage in the non-pT0 group, an organ-confined tumor (pT2) was found in 46 cases (43.8 %), locally-advanced disease including pT3 and pT4 were found in 46 cases (43.8 %), and pelvic lymph node (LN) metastasis was found in 13 cases (12.4 %). A positive surgical margin was observed in 28 patients (26.7 %) in the non-pT0 group including 7 patients (15.2 %) with stage pT2 and 15 patients (32.6 %) with stage pT3-pT4. ADT was added as an adjuvant treatment following RP in 18 patients (17.1 %), and there was no case that received adjuvant RT in this group. Patients in the pT0 group did not receive any additional treatment.

**Fig. 1:**
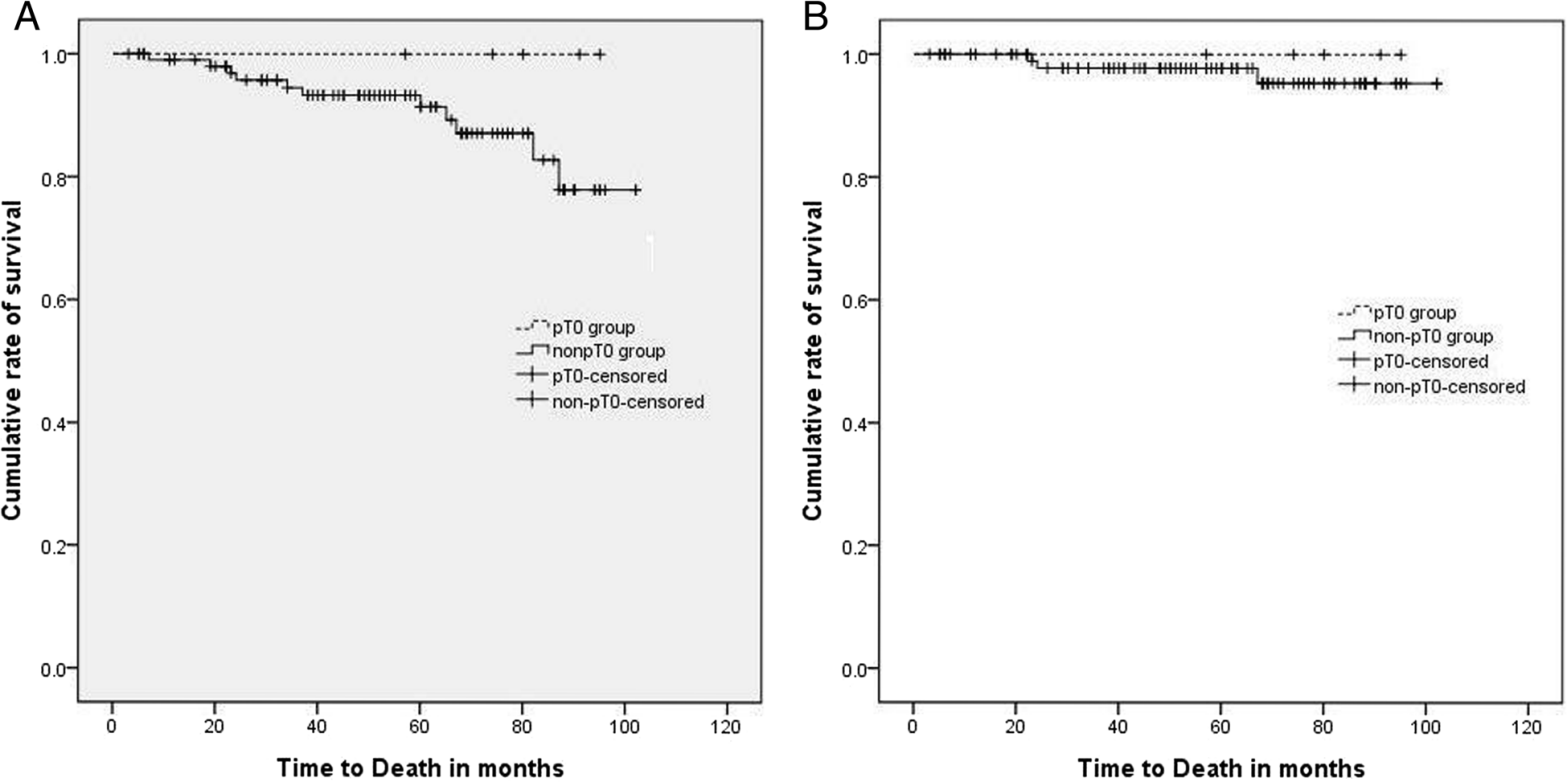
Overall survival (**a**) and cancer-specific survival (**b**) between the pT0 and non-pT0 groups

At the time of analysis, the median follow-up period was 54 months (range, 3 to 102 months) in all patients. In the pT0 group, all patients were alive and there was no recurrence of disease during the median follow-up of 59 months (range, 6 to 80 months). Among the 105 patients in the non-pT0 group, BCR developed in 60 patients (57.1 %), and the median time to BCR was 14 months (range, 5–56 months). Clinical progression occurred in 11 patients (10.5 %) including 9 patients (8.6 %) with local recurrence and 9 patients (8.6 %) with metastasis. 5-year PFS was 30.3 %, and 5-year clinical PFS and 5-year metastasis-free survival were 86.2 and 87.2 %, respectively. 6 patients (5.7 %) progressed to castration-resistant prostate cancer. Death occurred in 11 patients (10.5 %); prostate cancer-specific death in 3 cases (2.9 %) and death due to other causes in 8 cases (7.6 %). At 5 years, the overall survival (OS) and cancer-specific survival (CSS) were 91.5 and 97.7 %, respectively (Fig. 1).

## Discussion

In patients with high-risk prostate cancer, we observed 6 cases (5.4 %) of pT0 disease that showed no tumor recurrence during the follow-up. Our results indicate that ADT can induce a complete pathological response, and can potentially eradicate advanced prostate cancer.

Regarding the issue of pT0 disease in surgical specimens, the majority of surgeons were initially concerned about unnecessary surgery or overtreatment in patients with no residual tumors. The occurrence of pT0 disease has been reported in many previous studies of prostatectomy series. The prevalence of pT0 disease was found to be between 0.2 and 1.3 % in the prostatectomy series, and it was more common in localized prostate cancer in the low-risk group and in small tumors such as those with less than 2 positive cores on biopsy [1, 8–10]. In Korean patients, the likelihood of occurrence of pT0 disease was found to be related to the biopsy Gleason score, the number of positive cores on biopsy, the tumor length in a positive core, and prostate volume [10].

pT0 disease in prostatectomy specimens is more commonly detected in patients who receive ADT prior to surgery. Due to the effect of androgen deprivation, the prostate tumor shows glandular shrinkage including pyknosis, empty glandular spaces, and vacuolization and degeneration of tumor cells. In addition, ADT reduces the frequency of tumor extension to surgical margins [11].

The occurrence of pT0 disease following ADT has led to increased clinical interest in investigating the role of ADT, because patients with pT0 stage prostate cancer are expected to have an extremely favorable prognosis. NHT is initially intended to improve the surgical outcomes by minimizing the rate of positive surgical margins following RP. Early studies assessing the efficacy of NHT demonstrated a decrease in tumor volume and positive surgical margin rates, and an increase in the rate of pathologically organ-confined cancer [12, 13]. Many investigators have assessed the effect of combined NHT and RP on the survival outcomes in patients with prostate cancer, however, the effect of NHT adjunctive to RP has not been satisfactorily supported by existing evidence [3], which is different to the results seen with ADT prior to RT [14, 15]. Several guidelines have stated that there is no benefit to the administration of ADT prior to RP in localized cancer, based on the results of previous studies. Considering only high-risk or locally advanced prostate cancer, combined NHT and RP achieves a long-term PFS and OS comparable to the alternative strategy of combined RT and ADT [4, 5]. In the current study, we also achieved good results for the 5-year survival outcome, which was comparable to previous reports, despite a higher BCR rate.

With respect to the prevalence of pT0 disease after ADT, it is more common and has a wider prevalence compared with that in non-ADT cases. In one study, 36 (20.7 %) out of 174 patients who received NHT did not show any residual tumor in RP specimens [6]. In another study of high-risk disease, 2 (9.5 %) out of 21 patients did not show any residual tumor after 8 months of ADT before RP [7]. In the current study, pT0 disease was observed in 5.4 % of the patients with high-risk prostate cancer. This difference in the prevalence of pT0 after ADT may be related to the varying degree of diagnostic accuracy in detecting residual tumors that undergo histological changes following ADT, varying durations of ADT, and highly heterogeneous characteristics of tumors in each study.

In prostate cancer, diagnosis is generally made based on a routine pathological exam using standard processes such as H&E staining in a limited number of slides. In one study, after microscopic reassessment of the slides, very small tumor remnants with a mean volume of 0.2 ml were detected in 13 of the 20 prostatectomy specimens [16]. Considering our diagnostic procedure of using whole mounted sections and reexamination by pathologists who have excellent experience in the assessment of ADT-related cases, we are confident that the pT0 stage indicates a complete pathological response and eradication of the tumor following ADT.

The duration of ADT could also affect the incidence of pT0 disease. Prolonged NHT results in an additional decrease in tumor volume and positive surgical margin rates, and an increase in the rate of pathologically-confirmed organ-confined cancer and prevalence of pT0 disease [16, 17]. Based on the results of the current study, we found that ADT administered for only 3 months induced a complete pathological response in 2 patients.

The pathological response following ADT may be different depending on the tumor characteristics of each case. Among the prostatectomy cases that received NHT in a previous report, 43 % of patients still showed an unaltered area in tissues upon microscopic examination [11], indicating remarkable differences in the response of prostate cancer to ADT. In another previous study, after stratifying the rate of pT0 disease depending on the clinical stage (cT), pT0 was observed in 36.7 % of patients with cT1 prostate cancer, 33.9 % of patients with cT2 prostate cancer, and 5.9 % of patients with cT3 prostate cancer [6]. In a multi-institutional prospective trial including Japanese patients with high-risk disease, 8 months of ADT administration before RP resulted in no residual tumor (pT0) in 2 out of 21 patients (9.5 %) [7]. Despite being a retrospective study at a single institution, our present study included 111 patients with high-risk prostate cancer, of which 6 patients (5.4 %) were finally diagnosed as having pT0 disease, making this one of the largest data sets showing the rate of pT0 disease after NHT among patients in the high-risk group.

Theoretically, pT0 disease is assumed to have a good prognosis since relapse does not occur or occurs extremely rarely in pT0 patients. As expected, some studies have not shown biochemical or clinical progression of the disease [8, 18, 19]. However, Kollermann et al., showed that BCR was observed in 7 (18.4 %) of 38 patients with pT0 disease during a median follow-up of 47 months. Among these, 3 cases showed local or systemic tumor relapse [20]. Furthermore, PSA progression-free survival in the pT0 group was no different to that in the non-pT0 group in a matched pair analysis [6]. In a Japanese study of high-risk disease, BCR developed in half of the 21 patients with pT0 disease after 8 months of NHT [7]. These inconsistent results of clinical outcomes in pT0 disease are related to the diagnostic accuracy in detecting residual tumor and the length of follow-up. In fact, we cannot exclude the possibility that we may have experienced a relapse of the disease among patients with pT0 disease in the current study if the duration of the follow-up was longer. On the contrary, Bostwick et al., reported that none of the 34 pT0 patients had a relapse during a mean follow-up of 9.6 years, which is the longest follow-up data set to date [1].

Although we obtained encouraging results that showed no relapse of the disease, as well as the occurrence of pT0 disease in high-risk prostate cancer patients, there are some limitations to this study due to its retrospective nature.

Firstly, we only had 6 cases of pT0 disease. Although we analyzed some characteristics of the patients using a non-parametric method due to the very small number of pT0 patients, we could not compare the pT0 group with the non-pT0 group by performing a matched-pair analysis.

Secondly, the length of the follow-up period was limited. The median follow-up duration in the pT0 group was 59 months, which is not a sufficient time period to assess recurrence of the disease or the effect of pT0 disease on long-term survival in other localized prostate cancer with low or intermediate risk. Considering that BCR developed in more than 50 % of patients in the non-pT0 group of the current study, this follow-up duration may be sufficient to assess the clinical outcomes in high-risk prostate cancer.

Thirdly, there was no case of tumor relapse in the pT0 group. In some reports, pT0 patients have shown frequent recurrence of the tumor, which may be due to the probable over-diagnosis of pT0 disease due to insufficient partial sampling of prostatectomy specimens or missing small foci in the tumor. As already mentioned, we are confident that our pT0 stage indicates tumor eradication due to a complete pathological response to ADT, which led to good clinical outcomes of pT0 prostate cancer.

Although there was no statistically significant difference in the duration of ADT between the two groups, there was a wide variation in the duration of ADT due to the retrospective study design. It is assumed that the duration of NHT can affect the prevalence of pT0 disease and tumor relapse. Further prospectively-designed studies are required in order to refine and standardize the duration of therapy.

## Conclusions

Among the 111 patients with high-risk prostate cancer who received NHT and RP, we found 6 cases (5.4 %) that had no apparent residual tumor, and they were diagnosed as having pT0 disease from the prostatectomy specimens. Of interest, we observed that none of the pT0 patients showed disease recurrence, indicating a potential benefit of NHT as a preoperative treatment in patients with high-risk prostate cancer. Furthermore, NHT may have the ability to eradicate prostate cancer. This is one of the largest studies showing the prevalence of pT0 disease after NHT among patients with high-risk prostate cancer.

## References

[1] Bostwick DG, Bostwick KC (2004). ‘Vanishing’ prostate cancer in radical prostatectomy specimens: incidence and long-term follow-up in 38 cases. BJU Int.

[2] Cao D, Hafez M, Berg K, Murphy K, Epstein JI (2005). Little or no residual prostate cancer at radical prostatectomy: vanishing cancer or switched specimen?: a microsatellite analysis of specimen identity. Am J Surg Pathol.

[3] Hu J, Hsu J, Bergerot PG, Yuh BE, Stein CA, Pal SK (2013). Preoperative therapy for localized prostate cancer: a comprehensive overview. Maturitas.

[4] Berglund RK, Tangen CM, Powell IJ, Lowe BA, Haas GP, Carroll PR, Canby-Hagino ED, DeVere White R, Hemstreet GP, Crawford ED (2012). Ten-year follow-up of neoadjuvant therapy with goserelin acetate and flutamide before radical prostatectomy for clinical T3 and T4 prostate cancer: update on Southwest Oncology Group Study 9109. Urology.

[5] Powell IJ, Tangen CM, Miller GJ, Lowe BA, Haas G, Carroll PR, Osswald MB, De VWR, Thompson IM, Crawford ED (2002). Neoadjuvant therapy before radical prostatectomy for clinical T3/T4 carcinoma of the prostate: 5-year followup, phase ii southwest oncology group study 9109. J Urol.

[6] Kollermann J, Hopfenmuller W, Caprano J, Budde A, Weidenfeld H, Weidenfeld M, Helpap B (2004). Prognosis of stage pT0 after prolonged neoadjuvant endocrine therapy of prostate cancer: a matched-pair analysis. Eur Urol.

[7] Tabata K, Satoh T, Matsumoto K, Fujita T, Irie A, Iwamura M, Yanagisawa N, Matsuda D, Muramoto M, Kadowaki K (2006). 8 months of neoadjuvant hormonal therapy prior to radical prostatectomy for high-risk prostate cancer. Nihon Hinyokika Gakkai Zasshi.

[8] Trpkov K, Gao Y, Hay R, Yimaz A (2006). No residual cancer on radical prostatectomy after positive 10-core biopsy: incidence, biopsy findings, and DNA specimen identity analysis. Arch Pathol Lab Med.

[9] Mazzucchelli R, Barbisan F, Tagliabracci A, Lopez-Beltran A, Cheng L, Scarpelli M, Montironi R (2007). Search for residual prostate cancer on pT0 radical prostatectomy after positive biopsy. Virchows Arch.

[10] Park J, Jeong IG, Bang JK, Cho YM, Ro JY, Hong JH, Ahn H, Kim CS (2010). Preoperative clinical and pathological characteristics of pt0 prostate cancer in radical prostatectomy. Korean J Urol.

[11] Civantos F, Marcial MA, Banks ER, Ho CK, Speights VO, Drew PA, Murphy WM, Soloway MS (1995). Pathology of androgen deprivation therapy in prostate carcinoma. A comparative study of 173 patients. Cancer.

[12] van der Kwast TH, Tetu B, Candas B, Gomez JL, Cusan L, Labrie F (1999). Prolonged neoadjuvant combined androgen blockade leads to a further reduction of prostatic tumor volume: three versus six months of endocrine therapy. Urology.

[13] Gleave ME, La Bianca SE, Goldenberg SL, Jones EC, Bruchovsky N, Sullivan LD (2000). Long-term neoadjuvant hormone therapy prior to radical prostatectomy: evaluation of risk for biochemical recurrence at 5-year follow-up. Urology.

[14] Bolla M, de Reijke TM, Van Tienhoven G, Van den Bergh AC, Oddens J, Poortmans PM, Gez E, Kil P, Akdas A, Soete G (2009). Duration of androgen suppression in the treatment of prostate cancer. N Engl J Med.

[15] Bolla M, Gonzalez D, Warde P, Dubois JB, Mirimanoff RO, Storme G, Bernier J, Kuten A, Sternberg C, Gil T (1997). Improved survival in patients with locally advanced prostate cancer treated with radiotherapy and goserelin. N Engl J Med.

[16] Kollermann J, Feek U, Muller H, Kaulfuss U, Oehler U, Helpap B, Kollermann MW (2000). Nondetected tumor (pT0) after prolonged, neoadjuvant treatment of localized prostatic carcinoma. Eur Urol.

[17] Kollermann MW, Pantel K, Enzmann T, Feek U, Kollermann J, Kossiwakis M, Kaulfuss U, Martell W, Spitz J (1998). Supersensitive PSA-monitored neoadjuvant hormone treatment of clinically localized prostate cancer: effects on positive margins, tumor detection and epithelial cells in bone marrow. Eur Urol.

[18] Descazeaud A, Zerbib M, Flam T, Vieillefond A, Debre B, Peyromaure M (2006). Can pT0 stage of prostate cancer be predicted before radical prostatectomy?. Eur Urol.

[19] Herkommer K, Kuefer R, Gschwend JE, Hautmann RE, Volkmer BG (2004). Pathological T0 prostate cancer without neoadjuvant therapy: clinical presentation and follow-up. Eur Urol.

[20] Kollermann J, Caprano J, Budde A, Weidenfeld H, Weidenfeld M, Hopfenmuller W, Helpap B (2003). Follow-up of nondetectable prostate carcinoma (pT0) after prolonged PSA-monitored neoadjuvant hormonal therapy followed by radical prostatectomy. Urology.

